# A quantum heat engine driven by atomic collisions

**DOI:** 10.1038/s41467-021-22222-z

**Published:** 2021-04-06

**Authors:** Quentin Bouton, Jens Nettersheim, Sabrina Burgardt, Daniel Adam, Eric Lutz, Artur Widera

**Affiliations:** 1 Department of Physics and Research Center OPTIMAS, Technische Universität Kaiserslautern, Kaiserslautern, Germany; 2 Institute for Theoretical Physics I, University of Stuttgart, Stuttgart, Germany

**Keywords:** Nanoscale devices, Ultracold gases, Quantum physics, Thermodynamics

## Abstract

Quantum heat engines are subjected to quantum fluctuations related to their discrete energy spectra. Such fluctuations question the reliable operation of thermal machines in the quantum regime. Here, we realize an endoreversible quantum Otto cycle in the large quasi-spin states of Cesium impurities immersed in an ultracold Rubidium bath. Endoreversible machines are internally reversible and irreversible losses only occur via thermal contact. We employ quantum control to regulate the direction of heat transfer that occurs via inelastic spin-exchange collisions. We further use full-counting statistics of individual atoms to monitor quantized heat exchange between engine and bath at the level of single quanta, and additionally evaluate average and variance of the power output. We optimize the performance as well as the stability of the quantum heat engine, achieving high efficiency, large power output and small power output fluctuations.

## Abstract (web summary)

Designing reliable nanoscale quantum-heat engines achieving high efficiency, high power and high stability is of fundamental and practical interest. Here, the authors report the realization of such a quantum machine using individual neutral Cs atoms in an atomic Rb bath, in which quantized heat exchange via inelastic spin-exchange collisions is controlled at the level of single quanta.

## Introduction

Most engines used in modern society are heat engines. Such machines generate motion by converting thermal energy into mechanical work^1^. Two central figures of merit of heat engines are efficiency, defined as the ratio of work-output and heat input, and power characterizing the work-output rate. Heat engines should ideally have high efficiency, large power output, and be stable, i.e., exhibit small power fluctuations. However, real thermal machines operate far from reversible conditions and their performance is thus reduced by irreversible losses^2,3^. At the same time, microscopic motors are exposed to thermal fluctuations and, at low enough temperatures, to additional quantum fluctuations, which are associated with random transitions between discrete energy levels. Both fluctuation mechanisms contribute to their instability^4,5^. An important issue is hence to design and optimize small heat engines in order to maximize both their performance and their stability^6^.

Nanoscopic heat engines have been implemented recently using a single trapped ion^7^ and a spin coupled to the single-ion motion^8,9^. Indications for quantum effects have been reported in a spin engine consisting of nitrogen-vacancy centers interacting with a light field^10^, and quantum heat engine operation has been shown in nuclear magnetic resonance^11,12^ and single-ion^9^ systems. These thermal machines are based on harmonic oscillators or two-level systems, and the baths mediating heat exchange are simulated by interaction with either laser fields^7–10^ or radiofrequency pulses^11,12^.

We here experimentally realize a quantum Otto cycle using a large quasi-spin system in individual Cesium (Cs) atoms immersed in a quantum heat bath made of ultracold Rubidium (Rb) atoms. Expansion and compression steps are implemented by varying an external magnetic field, changing the energy-level spacing of the engine and performing work^13^. Heat exchange between system and bath occurs via inelastic endoenergetic and exoenergetic spin-exchange collisions^14^. The increased number of internal engine states, compared to simple two-level systems, allows for high-energy turnover per cycle, while their finite number naturally limits power fluctuations due to saturation, in contrast to the unbounded spectrum of harmonic oscillators. We employ quantum control of the coherent spin-exchange process^15^ to control the direction of heat transfer between system and bath at the level of individual quanta of heat^14^, independently of the kinetic thermal state of the bath. The precise control of the spin states of both engine and bath effectively suppresses internal irreversible losses in individual collisions, and thus makes the quantum heat engine endoreversible. Endoreversible machines operate internally without dissipation, while (external) irreversible losses only occur via the contact with the bath^2,3^. They hence outperform fully irreversible engines and have played for this reason a central role in finite-time thermodynamics for forty years^2,3^. We note that quantum systems generally exhibit internal friction when their Hamiltonian does not commute at different times^16,17^. We additionally characterize the discrete quantum heat transfer at the level of individual quanta using full-counting statistics^18,19^ and monitor the population dynamics of the engine from single-atom and time-resolved measurements of the engine’s quasi-spin distribution along the cycle. We employ this system and techniques to evaluate and optimize the performance as well as the stability of the quantum heat engine, achieving high efficiency, large power output and small power output fluctuations.

## Results

### Components of the neutral-atom machine

**Fig. 1:**
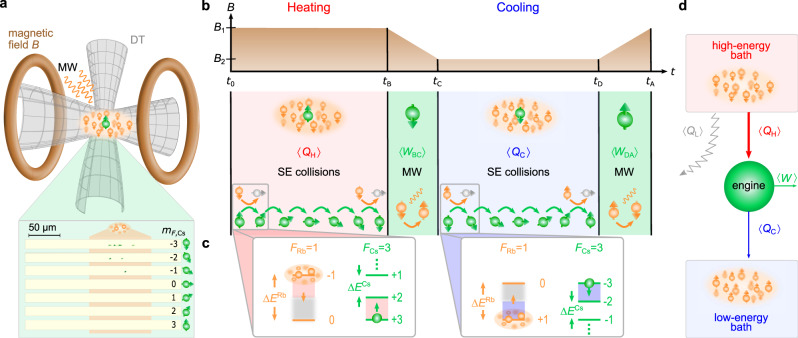
Operation principle of the quantum heat engine. **a** Individual laser-cooled Cs atoms (green) are immersed in an ultracold Rb cloud (orange); both are confined in a common optical dipole trap (DT). External magnetic fields and microwave (MW) radiation, respectively, implement the power strokes of the quantum heat engine and distinguish the high- from the low-energy bath. The inset shows typical *m*_*F*_-resolved fluorescence images of single Cs atoms for *t* = *t*_*B*_ = 300 ms after initialization, from which the quantized spin, and thus heat exchange, can be determined. The position of the bath cloud is indicated in orange with a width of 4*σ*. **b** The experimental Otto cycle consists of a heating stage, during which average heat 〈*Q*_H_〉 is absorbed, and a power stroke induced by an adiabatic change of the magnetic field. A microwave field then switches the bath from high to low energy. The cycle is further completed by a cooling step, during which average heat 〈*Q*_C_〉 is released, and an additional power stroke when the magnetic field is adiabatically brought back to its initial value. **c** The heat transfer between the Cs atom (engine) and a Rb (bath) atom occurs via inelastic spin-exchange collisions. In each collision, a single quantum of spin associated with a certain energy quantum is exchanged. Spin polarization of the Rb atoms and spin-conservation in individual collisions allow only up to six exo- or endothermal processes, corresponding to heating or cooling. **d** Owing to the difference of atomic Landé factors between Cs and Rb, the quantum heat engine (green) absorbs heat 〈*Q*_H_〉 and releases heat 〈*Q*_C_〉 (to produce work 〈*W*〉), while the bath releases more energy. The lost energy is irreversibly dissipated during an average of ten elastic collisions and is described by a heat leak 〈*Q*_L_〉 from the high-energy bath.

We experimentally immerse up to ten laser-cooled Cs atoms in the $|{F}_{\text{Cs}}=3,{m}_{F,\text{Cs}}=3\rangle$ state into an ultracold Rb gas of up to 10^4^ atoms in the state $|{F}_{\text{Rb}}=1,{m}_{F,\text{Rb}}=-1\rangle$, both species confined in a common optical dipole trap (Fig. 1a) (Methods A). Here *F* and *m*_*F*_ denote the total atomic angular momentum and its projection onto the quantization axis, respectively. The quantization axis is given by an external magnetic field of *B*_1_ = 346.5 ± 0.2 mG or *B*_2_ = 31.6 ± 0.1 mG. The Cs atoms quickly thermalize to the kinetic temperature of *T* = 950 ± 50 nK of the gas. We operate the quantum heat engine in the spin-state manifold of the seven Cs hyperfine ground states $|{F}_{\text{Cs}}=3,{m}_{F,\text{Cs}}\rangle$, *m*_*F*,Cs_ ∈ [+3, +2, …, −3], which define its quasi-spin. These states are energetically equally spaced with Zeeman energy ${E}_{n}^{\,\text{Cs}\,}=n\lambda B$, with $\lambda =| {g}_{F}^{\,\text{Cs}}| {\mu }_{\text{B}}$, where ${g}_{F}^{\,\text{Cs}\,}=-1/4$ is the Cs Landé factor, *μ*_B_ Bohr’s magneton and *n* = 3 − *m*_*F*,Cs_^20^, with the zero-point of energy set to the lowest-energy state $|{m}_{F,\text{Cs}}=3\rangle$.

Heat between the quantum engine and the bath is exchanged at the microscopic level via inelastic spin-exchange collisions (Fig. 1c). Each collision changes the value of the quasi-spin of the Cs engine by Δ*m*_Cs_ = ∓1*ℏ*, leading to an energy change of Δ*E*^Cs^ = ±*λB* for each Cs atom, and Δ*m*_Rb_ = ±1*ℏ* for one Rb atom corresponding to the energy change Δ*E*^Rb^ = ∓*κB*, with $\kappa =| {g}_{F}^{\,\text{Rb}}| {\mu }_{\text{B}}$, where ${g}_{F}^{\,\text{Rb}\,}=-1/2$ is the Rb Landé factor^14^. The spin population thus directly reflects the energy exchange between engine and reservoir at the level of single energy quanta. The direction of the heat transfer is determined by the spin polarization of the Rb bath and by angular momentum conservation during individual collisions^14^. The spin polarization of the Rb atoms distinguishes a high-energy bath for *m*_Rb_ = −1 from a low-energy bath for *m*_Rb_ = +1. Control over the internal Rb state accordingly permits to either increase or decrease the energy of the quasi-spin of the engine. Heat exchange automatically stops after six spin-exchange collisions, because then the highest/lowest energy state has been reached. One collision transfers the colliding Rb atom to the $|{F}_{\text{Rb}}=1,{m}_{F,\text{Rb}}=0\rangle$ state, which forms the exhaust of the engine. Owing to the massive imbalance between the Rb and Cs atom numbers (*N*_Rb_/*N*_Cs_ > 1000), the probability of a second collision with the same Rb atom is indeed vanishingly small. Memory effects are furthermore negligible, making the bath Markovian.

### Implementation of the quantum Otto cycle

The quantum Otto cycle consists of four parts: one compression and one expansion step, during which work is performed, and a heating and a cooling stage, during which heat is exchanged^13^. The corresponding experimental sequence is shown in Fig. 1b. The Cs machine is first driven by up to six spin-exchange collisions into energetically higher states (at magnetic field *B*_1_), absorbing average heat 〈*Q*_H_〉 in time *τ*_H_ = *t*_B_. Mean work 〈*W*_BC_〉 is then performed by adiabatically decreasing the magnetic field to *B*_2_ in *τ* = *t*_C_ − *t*_B_ = 10 ms. This time is much longer than the inverse energy splitting Δ*E* of the quasi-spin states, making the process adiabatic. It is, however, fast enough to avoid unwanted spin-exchange collisions, implying that no heat is transferred. The engine is subsequently brought into contact with the low-energy bath by flipping the spins of the Rb bath using microwave (MW) sweeps. The Cs engine is accordingly driven by up to six spin-exchange collisions into energetically lower states, releasing heat 〈*Q*_C_〉 in time *τ*_C_ = *t*_D_ − *t*_C_. Work 〈*W*_DA_〉 is further performed by adiabatically increasing the magnetic field back to *B*_1_ in *τ* = *t*_A_ − *t*_D_ = 10 ms. The Rb spins are finally flipped to their initial state with other microwave sweeps, restoring the high-energy bath.

**Fig. 2:**
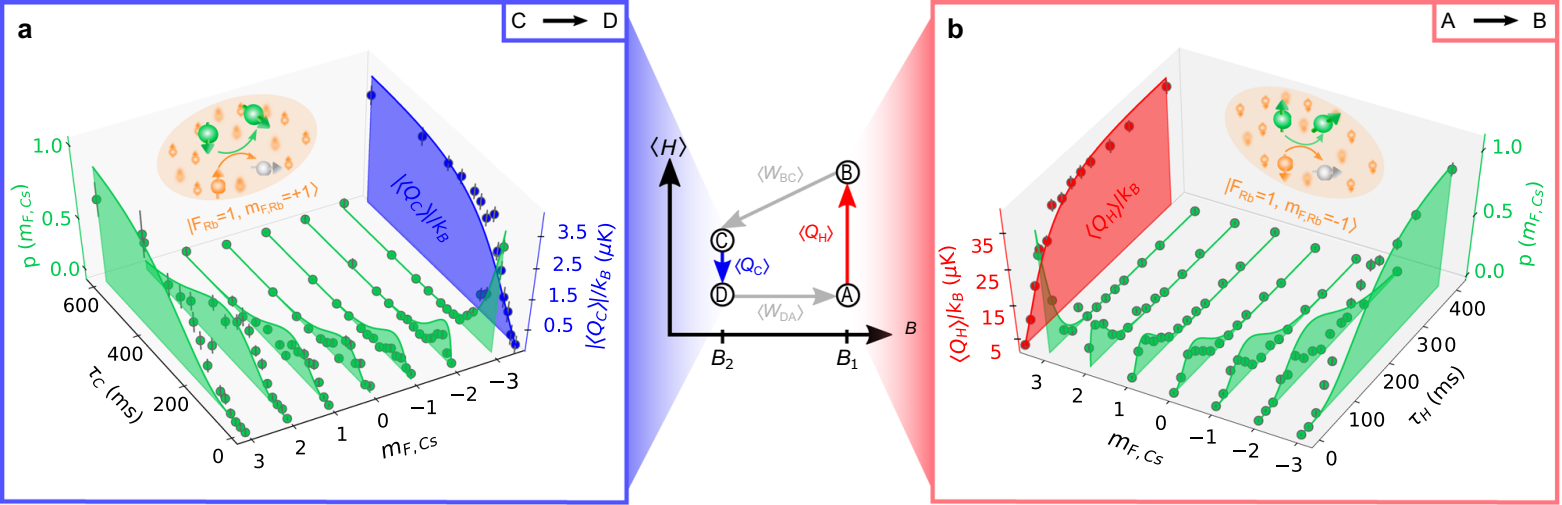
Full-counting statistics of heat exchange. During the heating (AB) and cooling (CD) steps of the quantum Otto cycle (center), heat is exchanged with the bath. The average population dynamics of the individual engine levels are shown in green. The mean heats, 〈*Q*_C_〉 and 〈*Q*_H_〉, extracted from the full-counting statistics are indicated for **a** cooling (blue) and **b** heating (red), as a function of the respective times *τ*_C_ and *τ*_H_. Dots show the experimental data, solid lines are a prediction of a microscopic model (Methods). In both panels, the population dynamics shows the transition from an initially spin-polarized engine state via a state of many populated *m*_*F*_ levels to a spin-polarized state of the other extreme spin state. The inversion of an initially fully polarized population ($|{m}_{F,\text{Cs}}=3\rangle \leftrightarrow |{m}_{F,\text{Cs}}=-3\rangle$) requires some hundreds of milliseconds. Error bars show statistical uncertainty of ± 1*σ* standard deviation.

While each single collision is coherent and thus amenable to quantum control^15^, coupling of the engine to the large number of bath modes in elastic collisions destroys the coherence between the engine’s quasi-spin levels. Heat is thus associated with changes of occupation probabilities, 〈*Q*〉 = ∑_*n*_*E*_*n*_Δ*p*_*n*_, whereas work corresponds to changes of energy levels, 〈*W*〉 = ∑_*n*_*p*_*n*_Δ*E*_*n*_^13^. In our system, we concretely have $\langle {Q}_{\text{H}}\rangle ={\sum }_{n}n\left({p}_{n}^{\,\text{B}}-{p}_{n}^{\text{A}\,}\right)\lambda {B}_{1}$ for heating and $\langle {Q}_{\text{C}}\rangle ={\sum }_{n}n\left({p}_{n}^{\,\text{D}}-{p}_{n}^{\text{C}\,}\right)\lambda {B}_{2}$ for cooling. On the other hand, the respective work contributions for expansion and compression are given by $\langle {W}_{\text{BC}}\rangle ={\sum }_{n}n{p}_{n}^{\,\text{B}\,}\lambda ({B}_{2}-{B}_{1})$ and $\langle {W}_{\text{DA}}\rangle ={\sum }_{n}n{p}_{n}^{\,\text{D}\,}\lambda ({B}_{1}-{B}_{2})$. In order to evaluate these average quantities, we determine the magnetic fields *B*_1_ and *B*_2_ with the help of Rb microwave spectroscopy (Methods). We further detect the Zeeman populations ${p}_{n}^{i}$ of single Cs atoms at arbitrary times by position resolved fluorescence measurements combined with Zeeman-state-selective operations (Fig. 1a inset)^21^. From each individual measurement, we can determine quantized spin transitions for each single atom. This allows us to monitor the resulting quantized heat exchange between engine and environment with a resolution of single quanta at each time. From a series of such measurements, we can further construct the average evolution of the quasi-spin populations (Fig. 2a, b): the progressive transfer from low (high) energy states to high (low) energy states during heating (cooling) as a function of time is clearly seen (green dots). From the measured heat counting statistics, we compute average (blue and red dots) and variance of heat exchange. We will use these quantities to examine the power output of the quantum machine and its fluctuations.

### Performance of the quantum heat engine

We first characterize the performance of the quantum Otto engine by evaluating its efficiency given by^13^,

1 $$\eta =\frac{\langle {Q}_{\text{H}}\rangle -| \langle {Q}_{\text{C}}\rangle | }{\langle {Q}_{\text{H}}\rangle +\langle {Q}_{\text{L}}\rangle },$$

where 〈*Q*_H_〉 − ∣〈*Q*_C_〉∣ is the total work produced by the machine, 〈*Q*_L_〉 the energy dissipated during the total heat exchange in one cycle, and 〈*Q*_H_〉 + 〈*Q*_L_〉 the heat emitted by the high-energy bath (Fig. 1d). Indeed, due to the different atomic Landé factors for Rb $({g}_{F}^{\,\text{Rb}\,}=-1/4)$ and Cs $({g}_{F}^{\,\text{Cs}\,}=-1/2)$, only half ($\gamma ={g}_{F}^{\,\text{Cs}}/{g}_{F}^{\text{Rb}\,}=1/2$) of the energy change of a bath atom is effectively exchanged with the heat engine during an inelastic spin-exchange collision^21^. As a result, the heat emitted (absorbed) by the bath differs from the energy portions absorbed 〈*Q*_H_〉 (emitted 〈*Q*_C_〉) by the machine. We macroscopically account for the remaining lost energy, which is irreversibly transferred to the kinetic energy of Rb during an average of ten elastic collisions, by a heat leak^22^ equal to $\langle {Q}_{\text{L}}\rangle ={\sum }_{n}n\left({p}_{n}^{B}-{p}_{n}^{A}\right)\kappa (1-\gamma )({B}_{1}-{B}_{2})$ with *γ* = *λ*/*κ* the ratio of the Landé factors (Methods). We then obtain,

2 $$\eta =\frac{\gamma ({B}_{1}-{B}_{2})}{{B}_{1}-{B}_{2}+\gamma {B}_{2}}\le 1-\frac{{B}_{2}}{{B}_{1}}={\eta }_{\text{max}}.$$

**Fig. 3:**
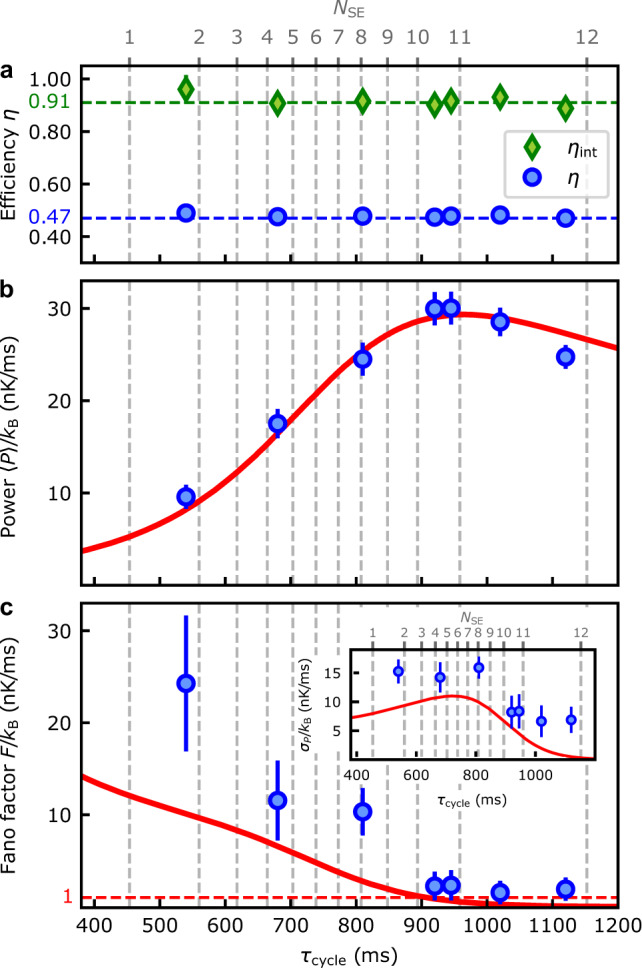
Performance of the quantum heat engine. **a** Efficiency *η*, Eq. (2) (blue dots), and internal (dissipationless) efficiency *η*_int_ (green diamonds) for different cycle times; dashed lines indicate the respective expected values. **b** Average power output, Eq. (3) (blue dots: experimental data, red solid line: theoretical model), with maximal value reached after almost 12 spin exchange collisions. **c** Fano factor, Eq. (4), and time-resolved fluctuations *σ*_*P*_ (inset). In all cases, the dashed vertical lines (upper axis) indicate the number of spin-exchange collisions *N*_SE_. The different durations between two successive spin-exchange collisions originate from different atomic transition rates^14^. Error bars show statistical uncertainty of ± 1*σ* standard deviation.

Its maximum value *η*_max_, reached in the absence of irreversible losses (*γ* = 1), is determined by the ratio of the two magnetic fields. We evaluate the efficiency (2) using experimental data for different cycle durations, *τ*_cycle_ = *τ*_H_ + *τ*_C_ + 2*τ*, by varying the heating and cooling times and evaluating the average heats 〈*Q*_H_〉 and 〈*Q*_C_〉 (Fig. 3a). We find a constant value, i.e., independent of the number of spin-exchange collisions, of *η* = 0.478 ± 0.002. We emphasize that the internal efficiency of the quantum Otto engine, *η*_int_ = 1 − ∣〈*Q*_C_〉∣/〈*Q*_H_〉 = 0.917 ± 0.009 (Methods C) is close to the maximal value *η*_max_ = 0.908. We may, therefore, conclude that irreversible losses mainly occur during heat transfer, while the engine itself runs reversibly. The quantum heat engine is hence endoreversible. We further note that, since heat losses are determined by the value of the Landé factors, they can in principle be reduced by choosing different atomic species.

Second, we consider the average power of the quantum heat engine which reads,

3 $$\langle P\rangle =\frac{\langle {Q}_{\text{H}}\rangle -| \langle {Q}_{\text{C}}\rangle | }{{\tau }_{\text{cycle}}}\le \frac{\langle {Q}_{\text{H}}\rangle }{{\tau }_{\text{cycle}}}\left(1-\frac{{B}_{2}}{{B}_{1}}\right).$$

We use the heat counting statistics to track its time evolution in Fig. 3b. We observe that the power (blue dots) increases with the number of inelastic collisions and reaches a maximum, ${\left\langle P\right\rangle }_{\max }/{k}_{\text{B}}=30$ nK/ms, for a cycle time of 960 ms. The corresponding number of inelastic collisions responsible for the heat exchange is almost 12 collisions total (6 spin-exchange collisions for the heating process and 6 for the cooling). This maximum nearly coincides with full population inversion between these two processes ($|{m}_{F,\text{Cs}}=3\rangle \leftrightarrow |{m}_{F,\text{Cs}}=-3\rangle$), in analogy to that of a laser. Good agreement with a theoretical model (red solid line) is observed (Methods). From a collisional perspective, the energy transfer with the atomic bath is optimal in the sense that it exchanges the maximum energy of six quanta, which can be stored in the machine, in exactly six spin-exchange collisions as a consequence of the precise control of the spin states of machine and bath. The value of ${\left\langle P\right\rangle }_{\max }$ may be further optimized by enhancing the magnetic field difference, as well as the collision rate and the collision cross-section by controlling the temperature or density of the Rb gas.

We finally investigate the stability of the quantum Otto engine by analyzing the relative power fluctuations via the Fano factor, which quantifies the deviation from a Poisson distribution^23^,

4 $${F}_{P}=\frac{{\sigma }_{P}^{2}}{\langle P\rangle }=\frac{\langle {P}^{2}\rangle -{\langle P\rangle }^{2}}{\langle P\rangle },$$

where ${\sigma }_{P}^{2}$ is the variance of the power, which we determine from the measured quasi-spin distributions (Methods). Figure 3c displays the Fano factor as a function of the cycle time, with the absolute fluctuations *σ*_*P*_ shown in the inset. We find super-Poissonian fluctuations (*F*_*P*_ > 1) for short cycle times, indicating that the quantum engine is unstable in this regime, with large relative power fluctuations. However, with increasing cycle time, the power increases faster than its variance, leading to a decrease in relative fluctuations. The transition to a Poissonian statistics (*F*_*P*_ = 1) (red dashed line), with strongly reduced power fluctuations and significantly increased stability, is located approximately at maximum power. This behavior follows from the finite Hilbert space of the Cs machine and the saturation effect due to the existence of an upper energy level. Importantly, the latter effect causes even the absolute value of the power fluctuations to decrease after on average six collisions (Fig. 3c inset). Power fluctuations could, in principle, also become sub-Poissonian (*F*_*P*_ < 1), but this regime is not seen experimentally due to experimental imperfections.

## Discussion

In conclusion, we have realized an endoreversible quantum Otto cycle using single Cs atoms interacting with a Rb bath. The key asset of this machine is the exquisite control over both the few-level engine and the atomic reservoir. This unique feature allows us not only to regulate and monitor the heat exchange between system and environment at the single-quantum level, but also to operate the quantum engine in a regime of high efficiency, large power output and small power output fluctuations. The produced work could in principle be extracted by, e.g., coupling the magnetic moment of another microscopic particle to the magnetic moment of the engine. In a magnetic field gradient, motion of the coupled microscopic particle will directly reveal the work performed. Our system provides a versatile experimental platform to elucidate fundamental new effects generated by quantum reservoir engineering, such as nonequilibrium atomic baths^24,25^ and squeezed baths^26,27^, as well non-Markovian heat reservoirs by reducing the size of the Rb cloud^28,29^.

## Methods

### Experimental procedures

We start our experimental sequence by preparing an ultracold Rb gas in the magnetic field insensitive state $|{F}_{\text{Rb}}=1,{m}_{F,\text{Rb}}=0\rangle$ and, at a distance of ≈200 μm, a small sample of laser-cooled Cs atoms. The Cs atoms are further cooled and optically pumped into the $|{F}_{\text{Cs}}=3,{m}_{F,\text{Cs}}=3\rangle$ hyperfine ground state by employing degenerate Raman sideband-cooling^30^. A species-selective optical lattice^31^ transports the Cs atoms into the Rb cloud. MW radiation prepares the bath atoms in the state $|{F}_{\text{Rb}}=1,{m}_{F,\text{Rb}}=-1\rangle$. The starting point of the heat engine cycle is defined by switching off the optical lattice potential. After a predefined time *t*_*i*_, the Cs-Rb interaction is stopped by freezing the positions of the Cs atoms using the optical lattice, and pushing the Rb cloud out of the trap with a resonant laser pulse. State-selective fluorescence imaging of the Cs atoms completes the procedure^32^.

**Fig. 4:**
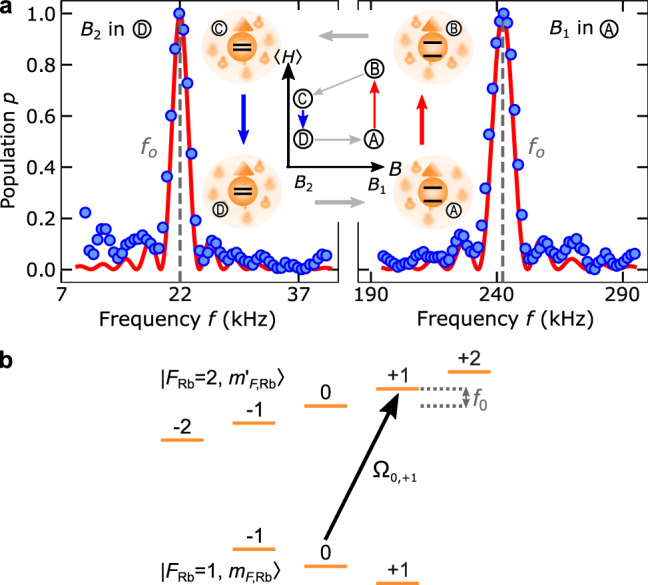
Magnetic field extraction. **a** Rb microwave spectra for extraction of the magnetic fields *B*_1_ and *B*_2_. Center illustrates the engine cycle and the corresponding Zeeman energy splitting of a Rb bath atom. Red lines correspond to the theory curves and blue dots are experimental data. These measurements yielding magnetic fields *B*_1_ = 346.5 ± 0.2 mG and *B*_2_ = 31.6 ± 0.1 mG. Measured spectra confirm similar magnetic fields for B and C. **b** Corresponding microwave transition scheme in the Rb ground-state hyperfine manifold.

The high-energy and low-energy baths are interchanged by transferring the Rb atoms from $|{F}_{\text{Rb}}=1,{m}_{F,\text{Rb}}=-1\rangle$ to $|{F}_{\text{Rb}}=1,{m}_{F,\text{Rb}}=+1\rangle$ and vice versa using two successive Landau–Zener sweeps. The transfer takes ~4.4 ms, which is fast enough to avoid spin-exchange interactions during the state change of the bath. The two magnetic fields *B*_1_ and *B*_2_ defining the quantization axis for the engine operation, are measured using Rb microwave spectroscopy on the $|{F}_{\text{Rb}}=1,{m}_{F,\text{Rb}}=0\rangle \to |{F}_{\text{Rb}}=2,{m}_{F,\text{Rb}}=+1\rangle$ transition. The population of the Rb atoms in state $|{F}_{\text{Rb}}=2,{m}_{F,\text{Rb}}=+1\rangle$ is detected by standard absorption imaging, using a time-of-flight measurement (Fig. 4). We fit the measured data with a standard model to extract the transition frequency, which translates into a magnetic field value using the Breit-Rabi formula^33^. We find typical errors of the order of 0.1 mG.

The magnetic field changes extracting work of the engine have to be adiabatic, i.e., preserving the populations *p*_*n*_. The adiabaticity condition writes $\dot{{\omega }_{\text{lar}}}/{\omega }_{\text{lar}\,}^{2}\ll 1$, where ${\omega }_{\text{lar}}=| {g}_{F}^{\text{Rb}}| {\mu }_{\text{B}}B/\hslash$ is the Larmor frequency. It can, therefore, be expressed as

5 $$A\equiv \frac{\hslash \dot{B}}{| {g}_{F}| {\mu }_{\text{B}}{B}^{2}}\ll 1.$$

Experimentally, we linearly vary the magnetic field from *B*_1_ = 346.5 ± 0.2 mG to *B*_2_ = 31.6 ± 0.1 mG in a time scale of 10 ms, yielding values of *A*(*B*_1_) = 0.2 × 10^−3^ and *A*(*B*_2_) = 14 × 10^−3^, thus fulfilling the adiabatic condition at any time during the variation of the magnetic field. Moreover, the time scale of the magnetic field variation is faster than the time scale associated with the spin exchange collisions (see number of collisions over time in Fig. 3). Hence, the populations *p*_*n*_ are constant during the isentropic processes (B → C and D → A).

### Microscopic model and number of collisions

The quantum heat exchange between engine and bath is based on the understanding of individual spin-exchange collisions. In general, the spin-collision rate ${{{\Gamma }}}^{{m}_{F}\to {m}_{F}\pm 1}$ is different both for every initial state *m*_*F*_ and for the direction, i.e., Δ*m*_*F*_ = ±1. The individual rates are well known from coupled-channel calculations of the molecular interaction potential between Rb and Cs^14^. These rates allow us to describe the evolution with a rate model^21^ that captures the spin dynamics and yields excellent agreement with the experimental data. From these rates, we also compute the mean number of spin collisions *N*_spin_ within a cycle duration *t* = *t*_D_ in two steps. First, we calculate the time-averaged collision rate as the sum of time-averaged collision rates during heating (exothermal spin collisions) and cooling (endothermal spin collisions) as

6 $$\left\langle \Gamma (t)\right\rangle 	=\left\langle {\Gamma }_{{\rm{A}}\to {\rm{B}}}(t)\right\rangle +\left\langle {\Gamma }_{{\rm{C}}\to {\rm{D}}}(t)\right\rangle \\ 	=\mathop{\sum }\limits_{{m}_{F}=+3}^{-2}{p}_{{m}_{F}}(t){\Gamma }_{{\rm{A}}\to {\rm{B}}}^{{m}_{F}\to {m}_{F}-1}\\ 	\quad+\mathop{\sum }\limits_{{m}_{F}=+2}^{-3}{p}_{{m}_{F}}(t){\Gamma }_{{\rm{C}}\to {\rm{D}}}^{{m}_{F}\to {m}_{F}+1}$$

Second, we integrate these rates during the heating and cooling to obtain the number of collisions within cycle time *t* as

7 $${N}_{\text{spin}}(t) 	={N}_{{\text{A}}\to \text{B}}+{N}_{{\text{C}}\to \text{D}}\\ 	=\int \nolimits_{0}^{{t}_{\text{B}}}(\langle {{{\Gamma }}}_{{\text{A}}\to \text{B}}({t}^{\prime})\rangle d{t}^{\prime}+\int \nolimits_{{t}_{\text{C}}}^{{t}_{\text{D}}}\langle {{{\Gamma }}}_{{\text{C}}\to \text{D}}({t}^{\prime})\rangle )d{t}^{\prime}.$$

In order to close the cycle, the inital and final Cs states before and after a cycle have to be the equal, leading to the condition *N*_A→B_ = *N*_C→D_.

### Efficiency of the endoreversibe machine

We calculate the efficiency by distinguishing two different forms of heat exchange. First, we consider the respective mean energies given (〈*Q*_1_〉) and taken (〈*Q*_2_〉) by the baths, where 〈*Q*_1_〉 − ∣〈*Q*_2_〉∣ is the energy turnover of the reservoirs per cycle. Second, we consider the average energies absorbed (〈*Q*_H_〉) and rejected (〈*Q*_C_〉) from the engine, where 〈*Q*_H_〉 − ∣〈*Q*_C_〉∣ is the energy turnover of the machine. Both quantities differ because of the different atomic Landé factors of Cs and Rb. The difference $\langle {Q}_{\text{L}}\rangle =\left(\langle {Q}_{1}\rangle -| \langle {Q}_{2}\rangle | \right)-\left(\langle {Q}_{\text{H}}\rangle -| \langle {Q}_{\text{C}}\rangle | \right)$ is dissipated via elastic collisions and irreversibly lost to the kinetic energy of Rb. We macroscopically model it as a heat leak from the high-energy reservoir. Using the population distribution of the quasi-spin levels at the cycle points in Fig. 2 of the main text, the individual heats can be calculated, leading to

8 $$\langle {Q}_{\text{L}}\rangle 	= \left(\langle {Q}_{1}\rangle -| \langle {Q}_{2}\rangle | \right)-\left(\langle {Q}_{\text{H}}\rangle -| \langle {Q}_{\text{C}}\rangle | \right)\\ 	= \left(\mathop{\sum}\limits _{n}n\left[{p}_{n}^{\,\text{B}}-{p}_{n}^{\text{A}\,}\right]\kappa {B}_{1}-\left|\mathop{\sum}\limits _{n}n\left[{p}_{n}^{\,\text{D}}-{p}_{n}^{\text{C}\,}\right]\kappa {B}_{2}\right|\right)\\ 	\quad -\left(\mathop{\sum}\limits _{n}n\left[{p}_{n}^{\,\text{B}}-{p}_{n}^{\text{A}\,}\right]\lambda {B}_{1}-\left|\mathop{\sum}\limits _{n}n\left[{p}_{n}^{\,\text{D}}-{p}_{n}^{\text{C}\,}\right]\lambda {B}_{2}\right|\right).$$

Owing to preservation of populations during adiabatic strokes, we can further use ${p}_{n}^{\,\text{D}}={p}_{n}^{\text{A}\,}$ and ${p}_{n}^{\,\text{B}}={p}_{n}^{\text{C}\,}$, yielding the expression for the dissipated heat

9 $$\langle {Q}_{{\rm{L}}}\rangle =\mathop{\sum}\limits _{n}n\left({p}_{n}^{B}-{p}_{n}^{A}\right)(\kappa -\lambda )({B}_{1}-{B}_{2}).$$

The efficiency is calculated as the work, ∣〈*W*〉∣ = 〈*Q*_H_〉 − ∣〈*Q*_C_〉∣, produced by the engine, divided by the energy provided by the high-energy bath, 〈*Q*_H_〉 + 〈*Q*_L_〉. Using ${p}_{n}^{\,\text{D}}={p}_{n}^{\text{A}\,}$, ${p}_{n}^{\,\text{B}}={p}_{n}^{\text{C}\,}$ and *γ* = *λ*/*κ*, we find

10 $$\eta =\frac{\langle {Q}_{\text{H}}\rangle -| \langle {Q}_{\text{C}}\rangle | }{\langle {Q}_{\text{H}}\rangle +\langle {Q}_{\text{L}}\rangle }=\frac{\gamma ({B}_{1}-{B}_{2})}{{B}_{1}-{B}_{2}+\gamma {B}_{2}}.$$

The internal efficiency of the engine is computed as the ratio of the produced work ∣〈*W*〉∣ and the heat absorbed by the machine 〈*Q*_H_〉:

11 $${\eta }_{{\rm{int}}}=\frac{\langle {Q}_{\text{H}}\rangle -| \langle {Q}_{\text{C}}\rangle | }{\langle {Q}_{\text{H}}\rangle }=1-\frac{{B}_{2}}{{B}_{1}}.$$

It corresponds to the efficiency without a leak (*γ* = 1).

### Fluctuations of the quantum machine

To extract the fluctuations of the engine, Eq. (4), we calculate the mean power, Eq. (3), via 〈*P*〉 = ∣〈*W*〉∣/*τ*_cycle_. The cycle time *τ*_cycle_ = *t*_D_ is experimentally controlled, and we assume that it is a fixed parameter not adding further fluctuations to the power-output fluctuations. Therefore, we can restrict the calculation to the fluctuations *σ*_*W*_ of work 〈*W*〉 as ${\sigma }_{W}^{2}=\langle {W}^{2}\rangle -{\langle W\rangle }^{2}$. The work is given by the difference of energy absorbed by and rejected from the engine ∣〈*W*〉∣ = 〈*Q*_H_〉 − ∣〈*Q*_C_〉∣, and hence

12 $${\sigma }_{W}^{2} 	={\sigma }_{{Q}_{\text{H}}}^{2}+{\sigma }_{{Q}_{\text{C}}}^{2}\\ 	 =\langle {Q}_{\,\text{H}\,}^{2}\rangle -{\langle {Q}_{\text{H}}\rangle }^{2}+\langle {Q}_{\,\text{C}\,}^{2}\rangle -{\langle {Q}_{\text{C}}\rangle }^{2}.$$

The averages and variances of heat absorbed or rejected depend on the energy differences at the different points during the cycle, for example, 〈*Q*_H_〉 = *E*(*t*_B_, *B*_1_) − *E*_0_(*t*_0_, *B*_1_). Here, $E({t}_{i},{B}_{j})={\sum }_{n}{p}_{n}^{i}({t}_{i})\ n\lambda {B}_{j}$ can be computed from the measured populations $\{{p}_{n}^{i}\}$ of level *n* at point *i* = A, B, C, D during the cycle and the magnetic field *B*_*j*_(*j* = 1, 2), together with mean energy and variance. Then, the fluctuations ${\sigma }_{Q}^{2}$ of heat 〈*Q*〉 exchanged when changing the engine’s probability distribution from point *i* to point *f* at a magnetic field *B*_*j*_ reads

13 $${\sigma }_{Q}^{2}	=\, \mathop{\sum}\limits _{n}\left({p}_{n}^{f}({t}_{f})+{p}_{n}^{i}({t}_{i})\right){(n\lambda {B}_{j})}^{2}\\ 	\quad-\left\{{\left[\mathop{\sum}\limits _{n}{p}_{n}^{f}({t}_{f})n\lambda {B}_{j}\right]}^{2}+{\left[\mathop{\sum}\limits _{n}{p}_{n}^{i}({t}_{i})n\lambda {B}_{j}\right]}^{2}\right\},$$

where, using the notation of Fig. 1b, for 〈*Q*_H_〉*i* = 0, *f* = B, and *B*_*j*_ = *B*_1_, and for 〈*Q*_C_〉*i* = C, *f* = D, and *B*_*j*_ = *B*_2_. Inserting these expressions into Eq. (12) allows us to compute the work fluctuations for every cycle time *τ*_cycle_ = *t*_A_ and thereby the variance of the output power fluctuations ${\sigma }_{P}^{2}$.

## Data Availability

The data that support the plots and findings of this study are available from the corresponding author upon reasonable request.
